# A Whole-Genome Survey and the Mitochondrial Genome of *Acanthocepola indica* Provide Insights into Its Phylogenetic Relationships in Priacanthiformes

**DOI:** 10.3390/ani14223257

**Published:** 2024-11-13

**Authors:** Weihua Mao, Ziyi Xu, Qi Liu, Na Li, Lu Liu, Biyan Ren, Tianxiang Gao, Chuan Liu

**Affiliations:** 1 Chongqing Key Laboratory of Big Data for Bio Intelligence, Chongqing University of Posts and Telecommunications, Chongqing 400065, China; 2 College of Resources and Environment, Southwest University, Chongqing 400716, China; 3 Fishery College, Zhejiang Ocean University, Zhoushan 316022, China

**Keywords:** Priacanthiformes, whole-genome survey, mitochondrial genome, genomic characteristics, phylogenetic evolution, population dynamics

## Abstract

*Acanthocepola indica*, a deep-sea snake fish, is primarily found in the Indo-west Pacific region, including India, Korea, Japan, and the South China Sea. The taxonomic classification of *A. indica* based on morphological characteristics remains inaccurate and unclear. In this study, we utilized next-generation sequencing to generate comprehensive genomic data for *A. indica*. The estimated genome size of *A. indica* was 422.95 Mb, with a heterozygosity ratio of 1.02% and a sequence repeat ratio of 22.43%. Our analysis suggested that *A. indica* is diploid, and the draft genome assembly consists of 1,059,784 contigs with a contig N50 of 1942 bp. We identified a total of 444,728 simple sequence repeats in the genome of *A. indica*. Furthermore, we successfully assembled the complete mitochondrial genome (16,439 bp) of *A. indica*, which included 13 protein-coding genes, 22 tRNA genes and 2 rRNA genes. Phylogenetic analysis based on mitochondrial genomes revealed that *A. indica* is closely related to *Acanthocepola krusensternii* and *Cepola schlegelii*, providing evidence that the family Cepolidae belongs to the order Priacanthiformes. Population size dynamics analysis indicated that *A. indica* experienced a bottleneck effect during the Pleistocene Glacial Epoch, likely due to the changes in glacial cycles and sea level fluctuations since ~800 Kya.

## Simple Summary

In this study, we explored the genetic characteristics and evolutionary history of *Acanthocepola indica*, a deep-sea snake fish. Genome sequencing revealed that *A. indica* is a diploid species with high heterozygosity and many repetitive sequences. We identified over 400,000 simple sequence repeats, which may serve as valuable markers for future genetic research. Additionally, we assembled the fish’s mitochondrial genome, uncovering important genes and patterns associated with amino acid production. Our analysis also showed that *A. indica* has experienced population declines, likely due to sea level changes during the Pleistocene Glacial Epoch. These findings lay the groundwork for further research on this species’ adaptation to deep-sea environments and support conservation efforts.

## 1. Introduction

*Acanthocepola indica*, a species of snake fish found at depths of approximately 300 m, is primarily distributed in the Indo-west Pacific region, encompassing India, Korea, Japan, and the South China Sea [1]. Due to the challenges associated with sampling deep-sea specimens, there have been limited records of this species. The most recent record of *A. indica* from Indian waters classified it within the Perciformes order [2]. However, according to the National Center for Biotechnology Information (NCBI) taxonomy database [3], *A. indica* is classified under the Priacanthiformes order, which includes two families, Cepolidae and Priacanthidae. The discrepancies in its classification may be attributed to the difficulty of defining any unique morphological characters for species in these orders. Hence, the application of genomic sequencing data and molecular markers could aid in clarifying the phylogenetic relationships of *A. indica*. Notably, there are currently no whole-genome sequencing data available for any species within the Priacanthiformes order.

With the advancement of high-throughput sequencing technologies, whole-genome data have become instrumental in studying genomic characteristics and developing high-polymorphism microsatellite markers in fish species [4,5,6,7]. Additionally, the assembly of mitochondrial genomes using next-generation sequencing (NGS) data allows for phylogenetic analysis [8,9,10,11,12,13], and historical population dynamics can be inferred from whole-genome data [14,15]. These genomic resources provide an invaluable resource for elucidating the genomic features and phylogenetic relationships of fish species with limited information.

This study employed NGS technology to acquire whole-genome data for *A. indica*, enabling the investigation of genomic characteristics and identification of microsatellites. The assembled mitochondrial genome was utilized for phylogenetic analysis among selected species of Priacanthiformes and Perciformes. Additionally, an analysis of historical population dynamics was performed to gain insights into the species’ evolutionary history and its responses to past environmental changes. The provision of genomic evidence for taxonomy and the advancement of evolutionary studies of fish species within the Priacanthiformes order will contribute significantly to the understanding of *A. indica* and related taxa.

## 2. Materials and Methods

### 2.1. Sample Collection and DNA Sequencing

The *A. indica* species was collected at the Beibu Gulf of the South China Sea. The samples were cryopreserved and transported to the Marine Fishery Resource and Biodiversity Laboratory of Zhejiang Ocean University. Preliminary morphological identification of the species was conducted following the methods outlined by Chen et al. [16]. Approximately 1 g of muscle tissue was collected for DNA extraction. The phenol/chloroform extraction method was used to extract the DNA from muscle tissue. The DNA concentration, purity and integrity were assessed using a NanoDrop 2000 (Thermo Fisher Scientific Inc, Waltham, MA, USA) and 1% agarose gel electrophoresis. After ultrasonic fragmentation, a library with insert fragment sizes of 300~400 bp was constructed for sequencing. The library was sequenced on the DNBseq platform according to the manufacturer’s protocol. The library construction and sequencing were performed at Wuhan Onemore-tech Co., Ltd., Wuhan, Hubei, China. The complete genomic sequencing data have been submitted to the Sequence Read Archive (SRA) database at the NCBI with accession number PRJNA1127248.

### 2.2. Genome Survey, Assembly and Simple Sequence Repeat (SSR) Identification

Fastp v0.23.2, with the length parameter “−l 50” and other default parameters, was used for raw data filtering [17]. Then, FASTQC v0.11.3 was used for clean read quality control by calculating the GC content and quality values of Q20 and Q30. To identify potential exogenous DNA contamination, the clean reads were aligned using the Basic Local Alignment Search Tool (BLAST) against the non-redundant (nr) protein sequence database. The nr database is a comprehensive collection of protein sequences from multiple sources, ensuring that our reads were compared against a wide range of known sequences to detect contamination. GCE v1.0.0 was used to estimate the genome characteristics with a K-mer size of 17 [18]. The outcomes of the K-mer analysis were leveraged to estimate the genome size, heterozygosity, and repeat ratio. Smudgeplot was applied to visualize and estimate the ploidy and structure of the *A. indica* genome by analyzing heterozygous k-mer pairs [19]. Minia v0.0.102, a short-read assembler based on a de Bruijn graph, was employed to assemble the clean reads into contigs [20]. The identification of potential SSRs was conducted using the Perl script “misa.pl” from the MISA v2.1 software with default parameters [21].

### 2.3. Mitochondrial Genome Assembly and Phylogenetic Analysis

The clean data were employed for the mitochondrial genome assembly and annotation using MitoZ v3.6 [22]. To clarify the phylogenetic relationships of *A. indica* within the order Priacanthiformes, available mitochondrial genome sequences for 8 species of Priacanthiformes and 7 species from other closely related orders were downloaded from the GenBank database (https://www.ncbi.nlm.nih.gov/), accessed on 15 May 2024. (Table 1). The mitochondrial genomes of all 16 species (including *A. indica*) were employed to reconstruct the phylogenetic tree, in which *Scortum barcoo* and *Tetraodon nigroviridis* were selected as outgroups. PhyloSuite v1.2.3 was applied for phylogenetic analyses, including sequence extraction, alignment, trimming, concatenation, and phylogenetic tree reconstruction [23]. Briefly, the nucleotide sequences of 13 protein-coding genes (PCGs), 22 tRNAs, and 2 rRNAs were first extracted. Then, the PCGs and RNAs were aligned and trimmed, respectively. After that, the trimmed PCGs and RNAs were concatenated and imported to ModelFinder for partitioning analysis. The maximum likelihood (ML) analysis was conducted using IQ-TREE integrated in PhyloSuite v1.2.3, which applied the automatically selected option of the model in IQ-TREE for 5000 ultrafast bootstrap replicates [24]. The final phylogenetic trees were viewed in iTOL v6.9 (https://itol.embl.de/, accessed on 15 May 2024) [25].

### 2.4. Effective Population Size Inferrence

In this study, the Pairwise Sequentially Markovian Coalescent (PSMC) method was employed for inferring the historical population dynamics of *A. indica*. The PSMC model estimates changes in effective population size over time by analyzing the distribution of heterozygous sites across the genome of a single diploid individual [26]. Specifically, clean reads were aligned to the assembled genome sequence using the BWA-mem method. Samtools (v0.1.19) was used to handle the mapped bam file using a parameter of “-bF 12” [27]. Then, bcftools and vcftools were used to convert the sorted bam files into “fq.gz” files, and the “fq2psmcfa” script in PSMC was used to convert the “fq.gz” file into a psmcfa file with a parameter of “-q20”. For running PSMC, the generation interval (g) was set to 1.5 years, and the mutation rate (μ) was set at 1.13 × 10^−9^ based on the result of Larimichthys crocea [28].

## 3. Results

### 3.1. The Genomic Estimation of A. indica

**Figure 1:**
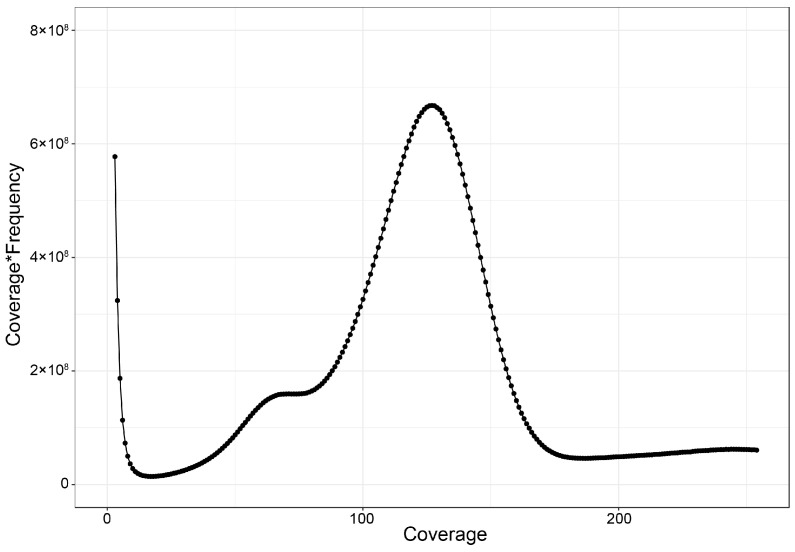
The genome size estimation of *A. indica* using K-mer (17-mer) analysis. The x-axis represents the sequencing depth, and the y-axis represents the frequency of k-mers. The peak indicates the estimated genome size and heterozygosity, providing insight into the overall genomic characteristics of the species.

**Figure 2:**
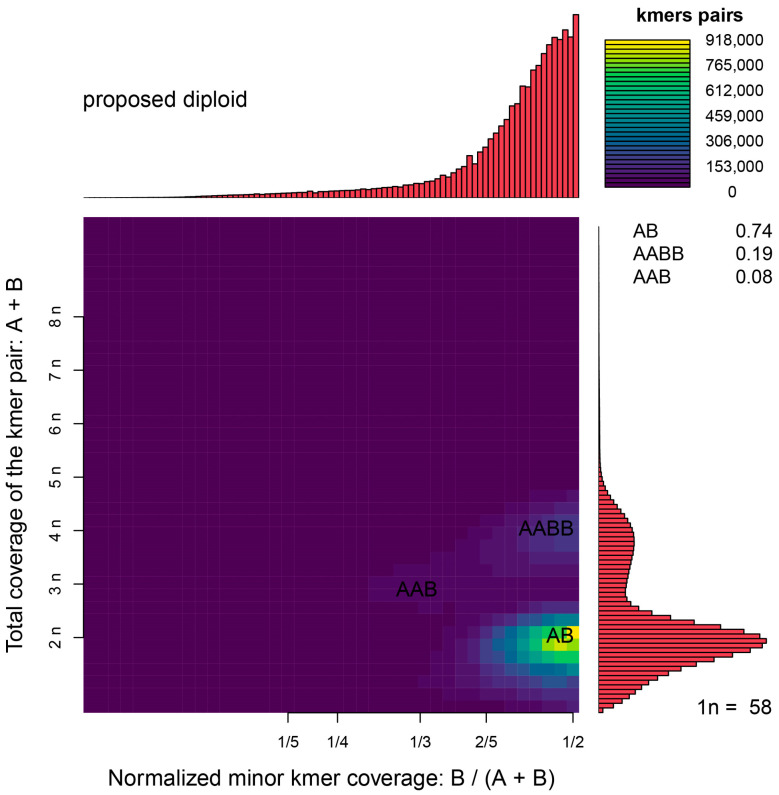
Genome ploidy level analysis of *A. indica*. The plot visualizes the heterozygous k-mer pairs, confirming that *A. indica* is a diploid species. The concentration of points within specific areas corresponds to diploid genome characteristics.

The sequencing of the *A. indica* library generated 68.37 Gb of raw data, consisting of approximately 455.83 M reads. The Q20, Q30 and GC contents of raw data were 96.00%, 88.49% and 44.00%, respectively (Table 2). A total of 67.14 Gb clean data were obtained by filtering the raw reads. The clean reads were compared to the NT database to detect DNA contamination, with the top three matched genera being *Lateolabrax*, *Sparus* and *Epinephelus* (Table S1), indicating no significant exogenous DNA contamination. K-mer analysis revealed a genome size of 422.95 Mb, a heterozygosity ratio of 1.02% and a sequence repeat proportion of 22.43% (Table 3). The 17-mer frequency plot showed high heterozygosity, with the highest peak at a depth of 124 (Figure 1). Smudgeplot analysis indicated that *A. indica* is a diploid species (Figure 2). The high heterozygosity and repetitive sequences present challenges for genome assembly [29,30], suggesting the need for higher sequencing depth, long-read sequencing and chromosome conformation capture technologies for future chromosome-scale genome assembly.

### 3.2. Genome Assembly and SSR Analysis

**Figure 3:**
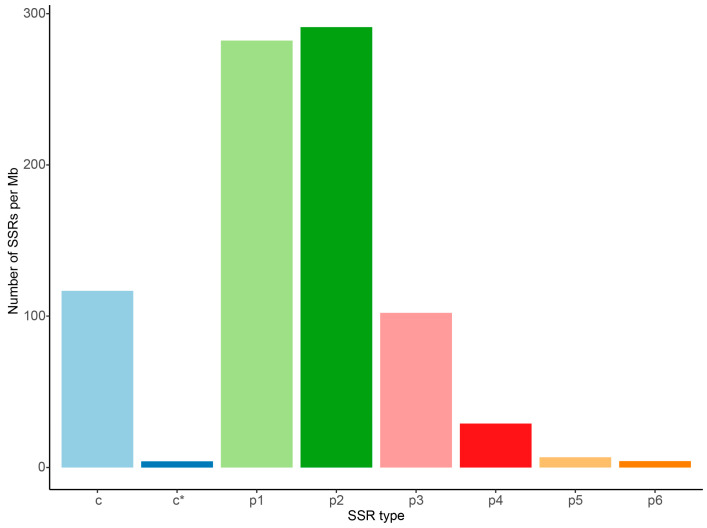
The frequency of microsatellite repeat types in *A. indica*. The figure illustrates the frequency distribution of different microsatellite repeat motifs identified in the assembled genome. The x-axis represents the length of SSR motifs, with p1 to p6 denoting mono-, di-, tri-, tetra-, penta- and hexa-nucleotide repeats, while “c” indicates compound SSRs and “c*” refers to compound SSRs where the different repeat types are located less than 100 bp apart. The y-axis shows the frequency of occurrence per megabase (Mb) of the genome.

The Minia v0.0.102 software was used to assemble the clean data into contigs, resulting in 1,059,784 contigs with a maximum length of 90,556 bp and a contig N50 length of 1942 bp (Table 4). The contig N50 length in this study is larger than that reported in several other fish draft genomes that also relied on NGS data [14,31]. The assembled genome size of *A. indica* was 531.79 Mb, accounting for 125.73% of the genome size assessed by 17-mer analysis. SSRs are commonly used in genetic diversity and evolutionary studies of fish species. Therefore, the MISA software was employed to identify SSRs from the assembled sequences. A total of 444,728 SSR sites were identified from the 531.79 Mb sequences, with an average of 836 SSRs per Mb. The SSR density, indicated by the number of SSRs per Mb, showed a decrease with increasing SSR unit length (Figure 3). For instance, mono-nucleotide (p1), di-nucleotide (p2) and tri-nucleotide (p3) exhibited 282, 291 and 102 SSRs per Mb, respectively. In contrast, the SSR density of tetra-nucleotide (p4), penta-nucleotide (p5) and hexa-nucleotide (p6) decreased significantly to 29, 7 and 4, respectively. Additionally, around 117 compound SSR (c) sites and 4 compound SSR sites with overlapping positions (c*) per Mb were identified from the assembled sequences (Figure 3). This finding aligned with previous studies revealing that dinucleotide SSRs are the most abundant [32,33]. These results may facilitate the identification of molecular markers, provide a genomic foundation for chromosome-scale genome assembly and further contribute to the population genetics of *A. indica*.

### 3.3. Characterization of A. indica Mitochondrial Genome

**Figure 4:**
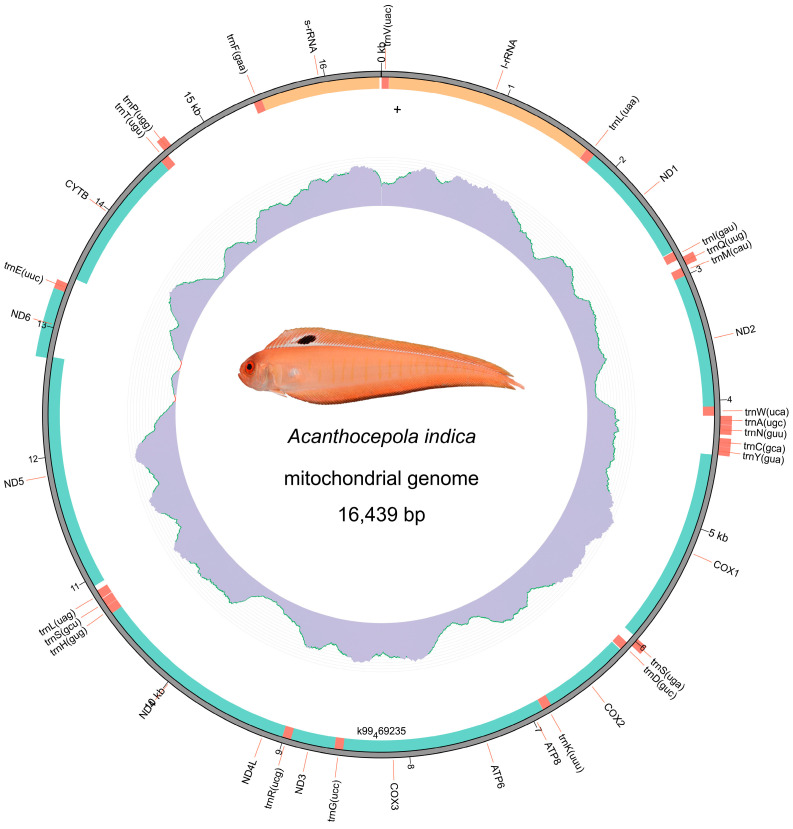
Mitochondrial genome organization of *A. indica*. The complete mitochondrial genome forms a circular molecule of 16,439 bp, comprising 37 genes: 13 protein-coding genes (PCGs), 22 tRNA genes, and 2 rRNA genes. Genes located on the heavy strand and light strand are labeled accordingly on the outer circle. The inner circle represents the sequencing depth across the mitochondrial genome, with fluctuations indicating variations in coverage throughout the genome.

**Figure 5:**
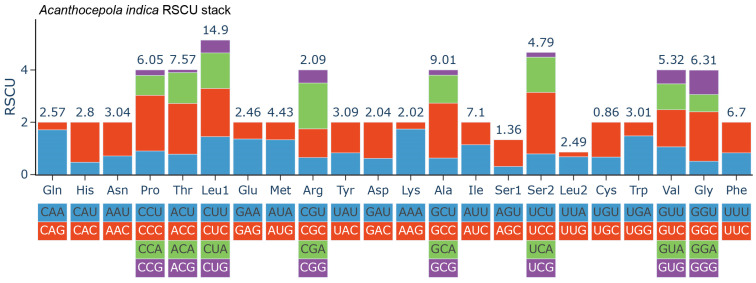
The relative synonymous codon usage (RSCU) for PCGs in the complete mitochondrial genome of *A. indica*. The different colors indicate different codon families corresponding to amino acids. The numbers above the bar plots represent the percentage of the amino acids for PCGs.

The complete mitochondrial genome of *A. indica* was assembled and annotated from the NGS clean data. The mitochondrial genome formed a closed circular molecule with a total length of 16,439 bp, comprising 37 genes, including 13 PCGs, 22 tRNA genes and 2 rRNA genes (Figure 4). Among these genes, 9 were distributed on the light strand, including ND6, trnE, tmP, tmQ, tmA, tmN, tmC, tmY and tmS, while the remaining 28 genes were located on the heavy strand (Table S2). All 13 PCGs, consisting of 7 NADH dehydrogenases, 3 cytochrome c oxidases, 2 ATP synthases and 1 cytochrome b, were identified and used to calculate the Relative Synonymous Codon Usage Calculation (RSCU) values (Figure 5). Most amino acids displayed codon usage bias towards specific codons, such as CAA (Gln), CAC (His), CCC (Pro) and others. Additionally, Pro, Thr, Leu, Ala, Ser, Val and Gly displayed relatively higher percentages (>5%), possibly due to being encoded by more codons (four or six) compared to the others. However, there was an exception, with Arg accounting for only 2.09% of PCGs. Conversely, Ile and Phe, encoded by only two codons, accounted for 7.1% and 6.7%, respectively. These results shed light on the codon and amino acid biases in the PCGs.

### 3.4. Phylogenetic Relationships of A. indica Based on Mitochondrial Genome

**Figure 6:**
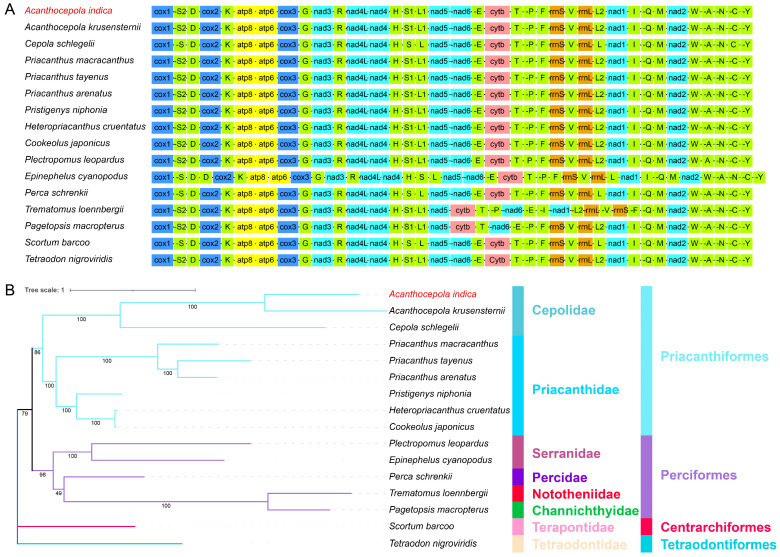
The phylogenetic tree reconstructed from the nucleotide sequences of thirteen PCGs, twenty-two tRNAs, and two rRNAs using IQ-TREE in PhyloSuite. (**A**) The gene orders of the concatenated nucleotide sequences for different species. (**B**) The family and order taxonomy of the fish species are indicated by different colored bars behind the phylogenetic tree. The numbers on the tree branches represent the bootstrap values.

Since we could not find any whole-genomic data for any species in the Priacanthiformes order in the NCBI Taxonomy database, we selected mitochondrial genomes of eight species from Priacanthiformes, five species from the closely related order Perciformes, one species from Centrarchiformes and one species from Tetraodontiformes for phylogenetic analysis (Table 1) in order to determine the phylogenetic relationships of *A. indica*. The two species from Centrarchiformes and Tetraodontiformes were used as outgroups. The phylogenetic tree was constructed using concatenated nucleotide sequences from all the PCGs, tRNAs and rRNAs (Figure 6A). The results showed that *A. indica* had a relatively close phylogenetic relationship with *Acanthocepola krusensternii*, a species in the same genus *Acanthocepola*. Additionally, *A. indica* was classified in the same clade as *Cepola schlegelii*, another species in the family Cepolidae (Figure 6B). Moreover, five other species from Priacanthidae formed a sister clade to Cepolidae, and both these clades belonged to Priacanthiformes. These findings further support previous phylogenetic relationships within the Priacanthiformes order [34]. Furthermore, five species of Perciformes formed another sister clade, in which *Perca schrenkii* exhibited a relatively close phylogenetic relationship with the clades of Priacanthiformes. This phylogenetic tree generally aligns with the NCBI taxonomy of fish species and provides the first insight into the phylogenetic relationships of *A. indica*.

### 3.5. Population Size Dynamics of A. indica

**Figure 7:**
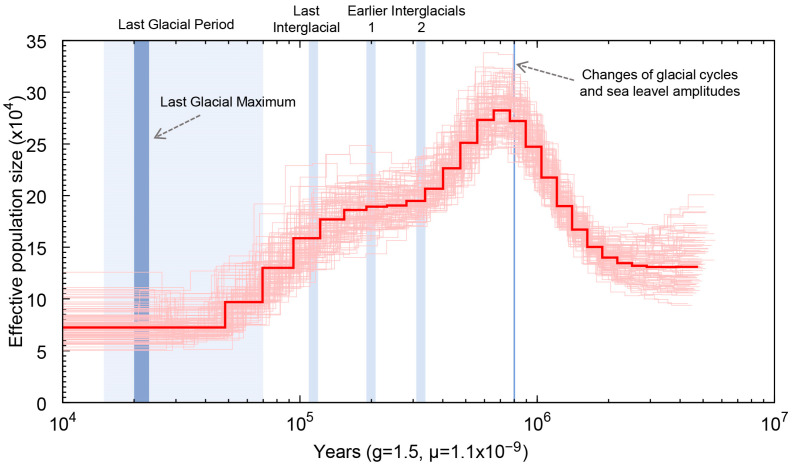
The demographic history of *A. indica* in this study. The PSMC estimates of demographic changes in effective population size over time were inferred from the draft genome sequences of *A. indica* in the present study. The thin light red lines indicate 100 rounds of bootstrapping results, and the thick red line represents the median values.

The PSMC model was employed to infer the historical changes in the effective population size of *A. indica* (Figure 7). The PSMC analysis indicated that *A. indica* experienced a bottleneck effect over the past million years. The effective population size of *A. indica* reached its peak approximately ~700 thousand years ago (Kya) and subsequently started to decline (Figure 7). The habitats of *A. indica*, predominantly located in the 300 m deep sea, may have been impacted by the changes in glacial cycles, with sea level amplitudes exceeding 100 m, that have occurred since ~800 Kya [35]. This alteration of their habitat likely posed a threat to their survival, resulting in a sharp decline in their population size since ~700 Kya. Interestingly, the decline in the effective population size slowed down between the two Earlier Interglacial periods (~330 Kya to ~200 Kya) [36] and then accelerated after the Last Interglacial period(~130–116 Kya). Eventually, the effective population size of *A. indica* reached a minimum during the Last Glacial period (~70–15 Kya), with no observable trend of recovery by ~10 Kya. Taken together, these results suggest that *A. indica* experienced a bottleneck effect during the Pleistocene Glacial Epoch. The changes in glacial cycles and sea level amplitudes (~800 Kya), along with the unstable climate of the Last Interglacial (~130–116 Kya), likely played major roles in the decrease in population size.

## 4. Discussion

Previous taxonomic research has primarily relied on morphological characteristics, such as the number of dorsal fin rays and body depth, to distinguish *A. indica* from other species like *A. limbata* and *A. krusensternii* [37,38,39,40]. *A. indica* was originally described as *Cepola indica* by Day in 1888 [41], and its classification has been refined over time through both morphological and genetic studies [2]. Although some genetic data are available, comprehensive genomic studies are still lacking. This study presented a draft genome of 531.79 Mb and a complete mitochondrial genome of 16,439 bp for *A. indica*. Our genomic data revealed that the *A. indica* genome was diploid, containing 444,728 simple sequence repeats. Phylogenetic analysis based on mitochondrial genomes confirmed that *A. indica* is part of the family Cepolidae, which belongs to the order Priacanthiformes. This finding not only underscores the taxonomic placement of *A. indica*, but also provides new insights into the genomic features and evolutionary relationships of the Cepolidae family within the Priacanthiformes.

Despite the advancements genomic sequencing has provided in understanding evolutionary relationships and genetic variability, our study, like others, was limited by the challenges in sampling deep-sea specimens. Consequently, a single specimen from specific geographic locations, primarily around India and East Asia, was used for recording phenotypic and genetic data [2,37,38,39,40]. This geographical bias may overlook the full extent of the species’ distribution and genetic diversity. Nevertheless, we successfully assembled the complete mitochondrial genome, comprising 37 genes, including 13 PCGs, 22 tRNA genes, and 2 rRNA genes (Figure 4). The gene distribution on heavy and light chains was consistent with *C. schlegelii* and other fish species [13,14,34]. Our RSCU analysis identified the codon and amino acid preference in PCGs, which could illuminate the genetic mechanisms and evolutionary pressures that shape codon usage.

Drops in the sea level led to a decrease in available coastal habitat and fragmented populations in many taxa, potentially resulting in high population genetic structuring [42]. For *A. indica*, which primarily inhabits depths around 300 m [16], sea level changes significantly impacted population size. Our analysis of population size dynamics indicated that *A. indica* experienced a bottleneck effect from approximately ~700 Kya to ~50 Kya, overlapping with the Pleistocene Glacial Epoch (Figure 7). This period coincided with significant changes in glacial cycles and sea level fluctuations, exceeding 100 m, since around ~800 Kya [35]. These findings suggest that sea level fluctuations played a critical role in reducing the population size of *A. indica*. Future research should focus on chromosome-scale genome assembly and population resequencing to gain a more detailed understanding of the genetic structure and evolutionary history of *A. indica*, potentially identifying specific genes responsible for adaptation to deep-sea environments.

## 5. Conclusions

This study presented novel findings on a whole-genome survey and a complete mitochondrial genome analysis of *A. indica*. The analysis revealed that the *A. indica* genome is diploid in nature and exhibits high heterozygosity. Additionally, the genome contained a significant number of repetitive DNA sequences. The draft genome of *A. indica* was successfully assembled, and more than four million SSR sites were identified. These findings will prove invaluable in facilitating future studies on the chromosome-scale genome assembly, comparative genome analysis and population genetics of *A. indica*. Furthermore, the complete mitochondrial genome of *A. indica* was assembled using NGS data, leading to the identification of biases in codons and amino acids for the PCGs. The phylogenetic tree constructed using the mitochondrial genomes confirmed that *A. indica*, as a member of the family Cepolidae, belongs to the order Priacanthiformes, reinforcing its taxonomic placement within this group. The PSMC analysis revealed that *A. indica* experienced a single bottleneck event during the Pleistocene Glacial Epoch, likely attributed to changes in glacial cycles and sea level amplitudes around ~800 Kya. This study not only offers a valuable data basis for chromosome-scale genome assembly, but also provides novel insights into resolving the phylogenetic relationships of *A. indica* within the Priacanthiformes order.

## Figures and Tables

**Table 1:** NCBI accession of mitogenomes of 16 species used in this study.

Species	Accession	Length (bp)	Order
*Acanthocepola indica*	PP962409	16,439	Priacanthiformes
*Acanthocepola krusensternii*	NC_034333.1	16,415	Priacanthiformes
*Cepola schlegelii*	NC_063676.1	17,020	Priacanthiformes
*Cookeolus japonicus*	NC_082750.1	16,506	Priacanthiformes
*Heteropriacanthus cruentatus*	NC_056807.1	16,506	Priacanthiformes
*Priacanthus arenatus*	NC_082997.1	16,996	Priacanthiformes
*Priacanthus macracanthus*	NC_029222.1	17,003	Priacanthiformes
*Priacanthus tayenus*	NC_029389.1	16,866	Priacanthiformes
*Pristigenys niphonia*	NC_031424.1	16,519	Priacanthiformes
*Epinephelus cyanopodus*	NC_068845.1	16,649	Perciformes
*Pagetopsis macropterus*	NC_057672.1	17,364	Perciformes
*Perca schrenkii*	NC_027745.1	16,536	Perciformes
*Plectropomus leopardus*	NC_008449.1	16,714	Perciformes
*Trematomus loennbergii*	NC_048965.1	19,374	Perciformes
*Scortum barcoo*	NC_027171.1	16,843	Centrarchiformes
*Tetraodon nigroviridis*	NC_031325.1	16,448	Tetraodontiformes

**Table 2:** The library sequencing statistics of *A. indica*.

Reads Type	Reads Number	Base Count (Gb)	Read Length (bp)	Q20 (%)	Q30 (%)	GC Content (%)
raw	455,825,818	68.37	150	96.00	88.49	44.00
dedup	448,476,588	67.14	149	96.01	88.49	44.00

**Table 3:** The K-mer-based genome survey result.

K-mer Number	K-mer Depth	Genome Size (bp)	Revised Genome Size (bp)	Heterozygous Ratio (%)	Repeat (%)
59,963,509,510	124	434,036,000	422,954,471	1.02	22.43

**Table 4:** Statistics of assembled genome in *A. indica*.

Total Number	Total Number (>2 kb)	Total Bases (bp)	Max Length (bp)	N50 (bp)	N90 (bp)	GC Content (%)
1,059,784	65,364	531,790,848	90,556	1942	168	43.5

## Data Availability

The raw genomic sequencing data presented in this study are publicly available under the BioProject accession number of PRJNA1127248. The mitogenome sequence data are openly available in GenBank of NCBI under accession no. PP962409.

## Supplementary Materials

The following supporting information can be downloaded at: https://www.mdpi.com/article/10.3390/ani14223257/s1, Table S1: BLAST result of sequencing reads to the NT database; Table S2: The organization of the complete mitochondrial genome of *A. indica*.
