# Supplementary material for: A Whole-Genome Survey and the Mitochondrial Genome of Acanthocepola indica Provide Insights into Its Phylogenetic Relationships in Priacanthiformes
Source: Animals (Basel). 2024 Nov 13;14(22):3257. doi: 10.3390/ani14223257 (PMC11590942; doi:10.3390/ani14223257)
Supplement: Supplementary file 1 [file animals-14-03257-s001.zip › animals-3247839-supplementary.pdf]

animals

MDPI

Article

Supplementary Materials

The Whole-Genome Survey and Mitochondrial Genome of *Acanthocepola indica* Provide Insights into its Phylogenetic Relationships in Priacanthiformes

Weihua Mao [1], Ziyi Xu [2], Qi Liu [1],Na Li [1], Lu Liu [1], Biyan Ren [1],Tianxiang Gao [3,*] and Chuan Liu [1,*]

1 Chongqing Key Laboratory of Big Data for Bio Intelligence, Chongqing University of Posts and Telecommunications, Chongqing 400065, China.
2 College of Resources and Environment, Southwest University, Chongqing 400716, China.
3 Fishery College, Zhejiang Ocean University, Zhoushan 316002, China.
* Correspondence: gaotianxiang0611@163.com (T.G.); liuchuan@cqupt.edu.cn (C.L.)

Table S1. BLAST result of sequencing reads to the NT database.

Species	Blast number	Total blast number	Total (%)
Lateolabrax	576	5,363	10.74
Epinephelus	507	5,363	9.45
Sparus	497	5,363	9.27
Nibea	414	5,363	7.72
Plectropomus	362	5,363	6.75
Larimichthys	330	5,363	6.15
Acanthopagrus	272	5,363	5.07
Sphaeramia	201	5,363	3.75
Cottoperca	200	5,363	3.73
Myripristis	174	5,363	3.24
Trachurus	155	5,363	2.89
Scophthalmus	75	5,363	1.4
Pseudochaenichthys	74	5,363	1.38
Anabas	73	5,363	1.36
Mastacembelus	70	5,363	1.31
Parambassis	68	5,363	1.27
Echeneis	64	5,363	1.19
Morone	62	5,363	1.16
Gouania	62	5,363	1.16
Dicentrarchus	59	5,363	1.1

Table S2. The organization of the complete mitochondrial genome of *A. indica*.

Gene Name	Gene Prodcut	Type	Start	End	Length (bp)	Strand
trnV	tRNA-Val (UAC)	tRNA	2	60	59	H
l-rRNA	16S ribosomal RNA	rRNA	61	1739	1679	H
trnL	tRNA-Leu (UAA)	tRNA	1740	1814	75	H
ND1	NADH dehydrogenase subunit 1	CDS	1814	2789	976	H
trnI	tRNA-Ile (GAU)	tRNA	2794	2864	71	H
trnQ	tRNA-Gln (UUG)	tRNA	2863	2934	72	L
trnM	tRNA-Met (CAU)	tRNA	2933	3002	70	H
ND2	NADH dehydrogenase subunit 2	CDS	3002	4049	1048	H
trnW	tRNA-Trp (UCA)	tRNA	4047	4120	74	H
trnA	tRNA-Ala (UGC)	tRNA	4120	4189	70	L
trnN	tRNA-Asn (GUU)	tRNA	4190	4263	74	L
trnC	tRNA-Cys (GCA)	tRNA	4289	4355	67	L
trnY	tRNA-Tyr (GUA)	tRNA	4355	4422	68	L
COX1	cytochrome c oxidase subunit I	CDS	4423	5974	1552	H
trnS	tRNA-Ser (UGA)	tRNA	5976	6047	72	L
trnD	tRNA-Asp (GUC)	tRNA	6051	6122	72	H
COX2	cytochrome c oxidase subunit II	CDS	6129	6828	700	H
trnK	tRNA-Lys (UUU)	tRNA	6820	6895	76	H
ATP8	ATP synthase F0 subunit 8	CDS	6896	7064	169	H
ATP6	ATP synthase F0 subunit 6	CDS	7054	7738	685	H
COX3	cytochrome c oxidase subunit III	CDS	7737	8523	787	H
trnG	tRNA-Gly (UCC)	tRNA	8522	8592	71	H
ND3	NADH dehydrogenase subunit 3	CDS	8592	8992	401	H
trnR	tRNA-Arg (UCG)	tRNA	8941	9011	71	H
ND4L	NADH dehydrogenase subunit 4L	CDS	9011	9308	298	H
ND4	NADH dehydrogenase subunit 4	CDS	9301	10682	1382	H
trnH	tRNA-His (GUG)	tRNA	10682	10751	70	H
trnS	tRNA-Ser (GCU)	tRNA	10752	10820	69	H
trnL	tRNA-Leu (UAG)	tRNA	10824	10896	73	H
ND5	NADH dehydrogenase subunit 5	CDS	10936	12778	1843	H
ND6	NADH dehydrogenase subunit 6	CDS	12763	13285	523	L
trnE	tRNA-Glu (UUC)	tRNA	13285	13355	71	L
CYTB	cytochrome b	CDS	13409	14550	1142	H
trnT	tRNA-Thr (UGU)	tRNA	14550	14622	73	H
trnP	tRNA-Pro (UGG)	tRNA	14622	14692	71	L
trnF	tRNA-Phe (GAA)	tRNA	15411	15479	69	H
s-rRNA	12S ribosomal RNA	rRNA	15479	16426	948	H
